# Association of Serial Intra-Abdominal Pressure Measurements with Renal Outcomes and Mortality in Critically Ill Adults

**DOI:** 10.3390/jcm15145742

**Published:** 2026-07-22

**Authors:** Ayşe Sarı Öztürk, Kamil Konur, Ekrem Kara

**Affiliations:** 1 Department of Internal Medicine, Gümüşhane State Hospital, Gümüşhane 29000, Türkiye; draysesarim@gmail.com; 2 Department of Internal Medicine, Division of General Internal Medicine, Faculty of Medicine, Recep Tayyip Erdogan University, Rize 53020, Türkiye; 3 Department of Internal Medicine, Division of Nephrology, Faculty of Medicine, Recep Tayyip Erdogan University, Rize 53020, Türkiye; ekrem.kara@erdogan.edu.tr

**Keywords:** intra-abdominal pressure, intra-abdominal hypertension, intensive care unit, acute kidney injury, abdominal compartment syndrome, mortality, critical illness

## Abstract

**Background/Objectives**: Intra-abdominal hypertension (IAH) is a common but under-recognized condition in critically ill patients and is associated with adverse outcomes, particularly acute kidney injury (AKI) and mortality. However, the prognostic value of intra-abdominal pressure (IAP) remains incompletely defined. This study aimed to evaluate the association between IAP levels and clinical outcomes, and to determine its prognostic significance in intensive care unit (ICU) patients. **Methods**: This prospective cohort study included adult patients admitted to the ICU between 2024 and 2025. Serial IAP measurements were performed using the transvesical method on days 0, 1, 3, 5, and 7. Demographic, clinical, and laboratory data, along with severity scores (SOFA and APACHE II), were recorded. Patients were followed for 90 days. Primary outcomes were AKI and 90-day mortality. Statistical analyses included regression models, survival analysis, and ROC curve analysis. **Results**: The prevalence of IAH was 54%. IAH was significantly associated with advanced age, male sex, higher body mass index, increased comorbidity burden, sepsis, positive fluid balance, and higher disease severity scores. Patients with IAH had a significantly higher frequency of AKI occurrence and increased mortality. IAP levels positively correlated with serum urea and creatinine levels. Serial IAP measurements, particularly on days 5 and 7, showed progressively improved predictive performance for mortality. Survival analysis revealed significantly reduced survival in patients with IAH. **Conclusions**: IAH is highly prevalent in critically ill patients and is strongly associated with renal dysfunction and mortality. Serial IAP measurements may provide useful prognostic information in critically ill patients; however, these findings require validation in larger studies.

## 1. Introduction

Intra-abdominal pressure (IAP) is defined as the steady-state pressure within the abdominal cavity. Under normal physiological conditions, IAP is typically below 4 mmHg and rarely exceeds 8 mmHg even in overweight individuals. The World Society of the Abdominal Compartment Syndrome (WSACS) defines intra-abdominal hypertension (IAH) as a sustained or repeated pathological elevation in IAP ≥ 12 mmHg [1].

The development of IAH is multifactorial and may occur in a wide range of clinical conditions, including trauma, major burns, abdominal surgery, severe heart failure, hepatorenal syndrome, and critical illness [2]. In addition, mechanical ventilation, reduced abdominal wall compliance related to body habitus and patient positioning, increased intra- and extraluminal abdominal contents, acidosis, sepsis, large-volume fluid resuscitation, pancreatitis, and increased capillary permeability may all contribute to elevated IAP. When these factors coexist, IAH may lead to significant morbidity and mortality if not promptly recognized and managed [3].

Acute kidney injury (AKI) is a clinical syndrome characterized by a rapid decline in renal function occurring over hours to days. It is typically identified by a decrease in glomerular filtration rate, an increase in serum creatinine levels, and/or reduced urine output. AKI can result in fluid and electrolyte imbalances, metabolic acidosis, accumulation of uremic toxins, and progression to multiple organ dysfunction.

When IAP exceeds 20 mmHg, abdominal compartment syndrome (ACS) may develop, leading to multi-organ failure and potentially death [2,4]. ACS represents the clinical manifestation of sustained IAH causing detrimental physiological effects on both abdominal and extra-abdominal organ systems [5,6]. Clinically, it is characterized by abdominal distension, respiratory compromise, and decreased urine output despite adequate fluid resuscitation [7,8].

The renal effects of IAH are primarily related to mechanical compression. Increased IAP leads to compression of the renal arteries, veins, and cortical arterioles, resulting in decreased renal blood flow, increased renal vascular resistance, and reduced glomerular filtration rate [9]. Additionally, activation of neurohormonal pathways, including increased levels of antidiuretic hormone, renin, and aldosterone, further exacerbates sodium and water retention [10]. These changes contribute to prerenal azotemia and may ultimately lead to the development of AKI.

Although ACS is a potentially reversible condition when recognized early and treated appropriately, delayed diagnosis and management may result in irreversible organ failure and death. Therefore, measurement of IAP plays a crucial role in identifying high-risk patients, preventing target organ damage, and reducing mortality in critically ill populations [11].

However, studies evaluating the relationship between intra-abdominal pressure and renal outcomes and mortality remain limited, and the available evidence is heterogeneous. In particular, the prognostic value of IAP in critically ill patients has not yet been clearly established. This gap highlights the need for further investigation into the clinical significance of IAP in intensive care settings.

In conditions such as IAH and ACS, abdominal perfusion pressure (APP) has emerged as an additional physiological parameter to assess the adequacy of organ perfusion. APP, defined as the difference between mean arterial pressure and intra-abdominal pressure, reflects the effective perfusion of abdominal organs, with values ≥ 60 mmHg generally considered adequate. Therefore, beyond isolated IAP measurements, complementary parameters such as APP may provide additional prognostic insight in critically ill patients. Low APP levels have been associated with organ hypoperfusion, renal failure, lactic acidosis, and increased mortality [12].

In this study, we aimed to evaluate the association between intra-abdominal pressure levels and demographic, clinical, and laboratory parameters in adult intensive care unit patients, and to investigate their impact on complications and mortality, as well as the prognostic value of IAP.

## 2. Materials and Methods

### 2.1. Study Design and Setting

This prospective cohort study was conducted in the adult intensive care unit (ICU) of Recep Tayyip Erdoğan University Training and Research Hospital between 2024 and 2025. The study included patients aged ≥18 years who were admitted to the ICU and followed for at least one week. The study protocol was approved by the Institutional Ethics Committee of Recep Tayyip Erdoğan University Faculty of Medicine (approval number: 2024/281) and conducted in accordance with the Declaration of Helsinki.

An a priori sample size calculation was performed using G*Power software (version 3.1.9.7; Heinrich Heine University Düsseldorf, Düsseldorf, Germany). The calculation was based on a chi-square test with one degree of freedom, assuming a medium effect size (Cohen’s w = 0.30), an alpha level of 0.05, and 80% statistical power. The minimum required sample size was calculated as 88 patients. Allowing for incomplete measurements and potential loss to follow-up, the target sample size was set at approximately 100 patients.

### 2.2. Study Population

Patients admitted to the ICU were prospectively screened for eligibility. Inclusion criteria were as follows: age ≥ 18 years, ICU admission for non-abdominal and non-cardiac surgical indications, absence of contraindications to transvesical IAP measurement, and availability of at least 7 days of follow-up data.

**Figure 1:**
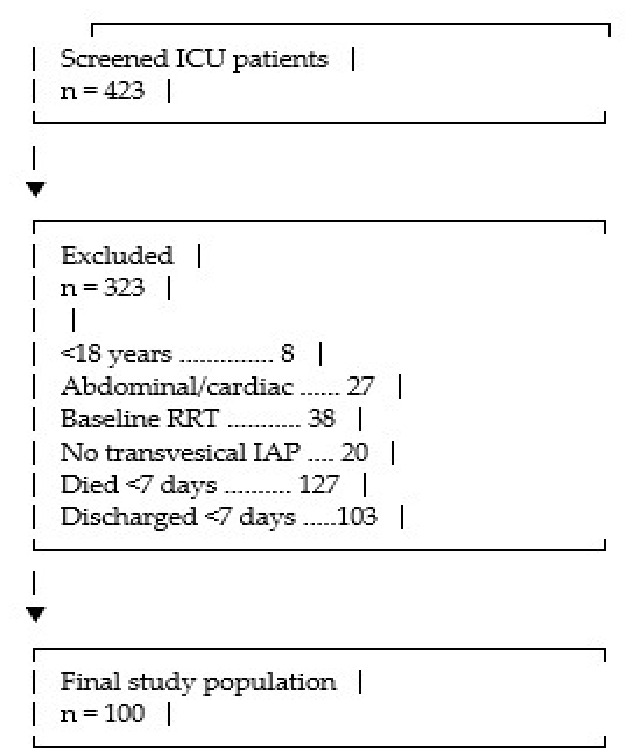
Flow Diagram of Patient Selection And Study Inclusion.

During the study period, 423 ICU patients were screened for eligibility. A total of 323 patients were excluded for predefined reasons, including age < 18 years, abdominal or cardiac surgery, baseline renal replacement therapy, inability to undergo transvesical IAP measurement, death within the first 7 days, or discharge within the first 7 days. The final study population consisted of 100 patients who completed the predefined serial intra-abdominal pressure measurements and were included in the primary analyses (Figure 1).

All patients were followed prospectively for 90 days. Mortality status was determined through review of the hospital electronic medical records and, when necessary, by telephone contact with the patients or their relatives. Complete 90-day follow-up data were available for all included patients, and no patients were lost to follow-up.

### 2.3. Data Collection

Demographic characteristics, comorbidities, causes of ICU admission, vital signs, and laboratory parameters were recorded for all patients. Disease severity scores, including the Sequential Organ Failure Assessment (SOFA) and Acute Physiology and Chronic Health Evaluation (APACHE II) scores, were calculated at baseline.

Clinical variables including volume status, fluid balance, and complications such as AKI, ACS, and sepsis were documented. Serial assessments of intra-abdominal pressure and laboratory parameters were performed on days 0 (baseline), 1, 3, 5, and 7 during ICU stay. Mortality status was recorded during the ICU stay and throughout the 90-day follow-up period.

### 2.4. Measurement of Intra-Abdominal Pressure

Intra-abdominal pressure was measured using the transvesical technique, which is widely accepted as a simple and cost-effective method. A bladder instillation volume of 20 mL sterile saline was used in accordance with current WSACS recommendations. This relatively small instillation volume minimizes bladder overdistension and has been shown to provide accurate and reproducible transvesical IAP measurements [13,14]. Patients were placed in the supine position, and measurements were performed under sterile conditions using a Foley catheter connected to a three-way stopcock system.

After clamping the drainage tubing, 20 mL of sterile saline was instilled into the bladder. The pressure transducer (or water manometer) was zeroed at the level of the symphysis pubis. To ensure standardization, all measurements were obtained at end-expiration, minimizing the effects of respiratory variation and mechanical ventilation.

IAH was defined as a sustained IAP ≥ 12 mmHg in accordance with current international consensus definitions.

### 2.5. Definitions and Outcomes

AKI was defined according to the Kidney Disease: Improving Global Outcomes (KDIGO) criteria as an increase in serum creatinine by ≥0.3 mg/dL within 48 h, an increase to ≥1.5 times the baseline value within 7 days, and/or urine output < 0.5 mL/kg/h for at least 6 h. Baseline serum creatinine was defined as the most recent stable creatinine value available prior to ICU admission, in accordance with the KDIGO recommendations. Urine output was monitored continuously through an indwelling urinary catheter as part of routine ICU care. AKI was assessed throughout the ICU stay, and patients were classified as having AKI if they fulfilled the KDIGO diagnostic criteria at any time during follow-up. Baseline AKI and incident AKI were not analyzed separately.

Assessment of volume status was performed at ICU admission based on clinical examination findings. Patients were categorized as hypovolemic, euvolemic, or hypervolemic according to the overall clinical assessment of the treating physician. Classification was based on physical examination findings, including peripheral edema, jugular venous distention, pulmonary auscultation findings, mucosal hydration status, blood pressure, and other clinical signs of volume overload or depletion. Fluid balance was assessed at admission using the available fluid intake and output records. Fluid management was performed according to standard ICU practice and individualized by the treating intensivists based on the patient’s hemodynamic status, urine output, laboratory findings, and overall clinical condition. No protocol specifically targeting intra-abdominal pressure was applied during the study period.

IAH was defined as an IAP ≥ 12 mmHg according to the WSACS criteria. The same diagnostic threshold was used for both admission IAH and follow-up IAH. Admission IAH was defined as IAP ≥ 12 mmHg at ICU admission (day 0), whereas follow-up IAH was defined as new-onset IAH occurring during follow-up in patients without IAH at admission. Patients were classified as having follow-up IAH if an IAP value ≥ 12 mmHg was recorded at any subsequent measurement point (days 1, 3, 5, or 7).

ACS was defined as a sustained IAP > 20 mmHg associated with new organ dysfunction.

The primary outcomes of the study were AKI occurrence during ICU follow-up and 90-day mortality. Secondary outcomes included the development of ACS and the association between IAP levels and clinical and laboratory parameters.

### 2.6. Statistical Analysis

Statistical analyses were performed using IBM SPSS Statistics version 27.0 (IBM Corp., Armonk, NY, USA). Continuous variables were expressed as mean ± standard deviation, and categorical variables were presented as frequencies and percentages. Normality of distribution was assessed using the Kolmogorov–Smirnov test.

Comparisons between two independent groups were performed using Student’s *t*-test for normally distributed variables and the Mann–Whitney U test for non-normally distributed variables. For comparisons among three or more groups, one-way ANOVA or the Kruskal–Wallis test was used, as appropriate. Categorical variables were compared using the chi-square test.

Logistic regression analysis was performed to identify factors associated with intra-abdominal hypertension. Survival analysis was conducted using the Kaplan–Meier method, and differences between groups were assessed using the log-rank test. Cox proportional hazards regression analysis was used to determine independent predictors of mortality.

Variables with a *p*-value < 0.10 in univariable analyses and variables considered clinically relevant were initially entered into full multivariable regression models. To reduce model complexity and limit overfitting, parsimonious final models were subsequently fitted using the predictors that remained independently associated with the outcome in the full models. Model complexity was evaluated by calculating the events-per-variable (EPV) ratio, defined as the number of outcome events divided by the number of predictor parameters included in the model. For logistic regression, model discrimination was assessed using the area under the receiver operating characteristic curve (AUC), and calibration was evaluated using the Hosmer–Lemeshow goodness-of-fit test and the calibration slope. For Cox regression, discrimination was evaluated using Harrell’s concordance index (C-index), and calibration was assessed using the calibration slope. Internal validation was performed using 1000 bootstrap resamples, and optimism-corrected AUC and C-index estimates were calculated.

Exploratory interaction analyses were performed to evaluate whether the associations of IAH with AKI and 90-day mortality differed according to sepsis, mechanical ventilation, age, and BMI. Results are presented in Supplementary Table S1.

Receiver operating characteristic (ROC) curve analysis was also performed to evaluate the diagnostic performance of serial intra-abdominal pressure measurements in predicting mortality. A *p*-value < 0.05 was considered statistically significant.

## 3. Results

The patient selection process is presented in Figure 1. Of 423 patients screened during the study period, 100 fulfilled all eligibility criteria and completed the predefined serial IAP measurements. These patients constituted the final study cohort.

A total of 100 adult ICU patients were included (mean age 71.3 ± 15.3 years, 56% male). IAP was within the normal range (<12 mmHg) in 46% of patients (*n* = 46), whereas 54% (*n* = 54) developed IAH. Among these, 24 patients had IAH at admission, while 30 developed IAH during the follow-up. Overall mortality was 57% (*n* = 57), while 43% (*n* = 43) survived.

### 3.1. Clinical Characteristics According to IAP Status

Patients with IAH were significantly older and had a higher burden of comorbidities compared to those with normal IAP. Disease severity scores (SOFA and APACHE II), the prevalence of sepsis, positive fluid balance, and the need for inotropic support were all significantly higher in the IAH group.

The incidence of AKI was markedly increased in patients with IAH. Notably, mortality was significantly higher in this group compared to patients with normal IAP (77.8% vs. 32.6%) (Table 1).

### 3.2. Baseline Laboratory Parameters Associated with Mortality

Non-survivors exhibited significantly higher levels of urea, creatinine, lactate dehydrogenase (LDH), procalcitonin, and lactate compared to survivors, while aspartate aminotransferase (AST) and pH levels were significantly lower. These findings indicate more pronounced metabolic derangement, systemic inflammation, and organ dysfunction in patients with poor outcomes (Table 2).

### 3.3. Correlation Analysis

The correlations between IAP and clinical/laboratory parameters are presented in Table 3. Correlation analysis demonstrated that IAP was positively correlated with BMI (r = 0.221, *p* = 0.027), CCI (r = 0.332, *p* = 0.001), APACHE II score (r = 0.397, *p* < 0.001), serum urea (r = 0.508, *p* < 0.001), creatinine (r = 0.672, *p* < 0.001), AST (r = 0.244, *p* = 0.015), ALT (r = 0.199, *p* = 0.048), LDH (r = 0.199, *p* = 0.048), procalcitonin (r = 0.410, *p* < 0.001), and lactate levels (r = 0.310, *p* = 0.002). Significant negative correlations were observed between IAP and albumin (r = −0.296, *p* = 0.003), pH (r = −0.299, *p* = 0.003), and bicarbonate levels (r = −0.300, *p* = 0.002).

### 3.4. Subgroup Analysis According to Timing of IAH Development

When patients were stratified into IAH at admission and IAH developing during the follow-up, both IAH groups demonstrated significantly worse clinical profiles. These patients exhibited higher comorbidity burden, increased sepsis rates, and elevated disease severity scores, along with significantly higher rates of AKI, need for renal replacement therapy, and mortality (Table 4).

### 3.5. Factors Associated with Intra-Abdominal Hypertension

In the parsimonious multivariable logistic regression model, higher SOFA score and the presence of AKI were independently associated with IAH. Each one-point increase in SOFA score was associated with a 1.54-fold increase in the odds of IAH (OR 1.54, 95% CI 1.17–2.01; *p* = 0.002), while AKI was associated with markedly increased odds of IAH (OR 29.44, 95% CI 8.50–102.00; *p* < 0.001). The model included 54 IAH events and two predictors, corresponding to an EPV of 27.0. Discrimination was high (AUC 0.922), and the optimism-corrected AUC after 1000 bootstrap resamples was 0.920. Calibration was acceptable (Hosmer–Lemeshow *p* = 0.296; bootstrap calibration slope = 0.938) (Table 5).

### 3.6. Multivariable Cox Regression Analysis of Factors Associated with Mortality

In the parsimonious Cox regression model, development of IAH and Charlson Comorbidity Index were independently associated with mortality. IAH was associated with a 2.48-fold higher mortality hazard (HR 2.48, 95% CI 1.32–4.68; *p* = 0.005), while each one-point increase in CCI was associated with a 15% increase in mortality hazard (HR 1.15, 95% CI 1.05–1.26; *p* = 0.003). The model included 57 deaths and two predictors, corresponding to an EPV of 28.5. The apparent Harrell C-index was 0.736 and the optimism-corrected C-index after 1000 bootstrap resamples was 0.733. The bootstrap calibration slope was 0.973, indicating minimal optimism (Table 6).

### 3.7. Exploratory Interaction Analyses

Exploratory interaction analyses showed no significant effect modification of the associations between IAH and AKI or 90-day mortality according to sepsis, mechanical ventilation, age, or BMI (all *p* for interaction > 0.05; Supplementary Table S1).

### 3.8. Survival Analysis

**Figure 2:**
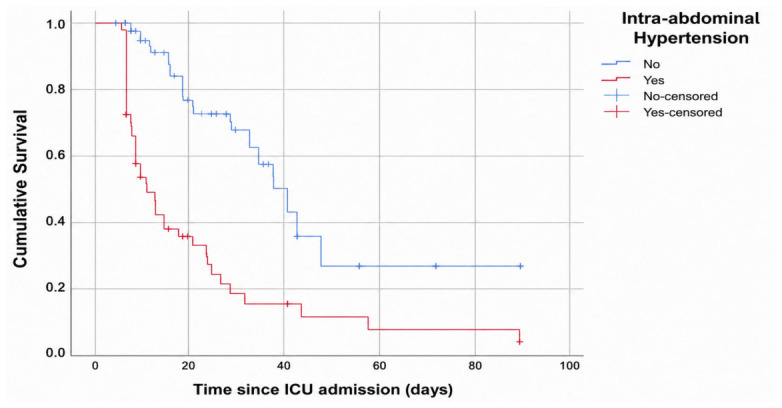
Kaplan–Meier Survival Curves For 90-Day Mortality According To Intra-Abdominal Hypertension.

Kaplan–Meier survival analysis demonstrated that the presence of IAH was associated with significantly reduced survival. Patients with IAH had shorter median survival compared to those without IAH, indicating a clear relationship between IAH and poor clinical outcomes. The separation between survival curves became apparent early in the follow-up period and continued to increase over time, suggesting that the negative impact of IAH begins in the early phase of critical illness and persists throughout the ICU stay. Overall, these findings support the role of IAH as an important prognostic factor associated with reduced survival in critically ill patients (Figure 2).

### 3.9. Diagnostic Performance of IAP

**Figure 3:**
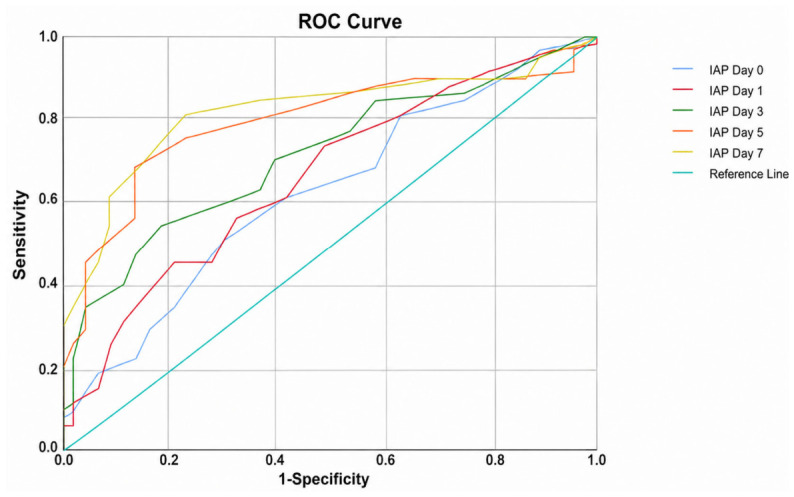
ROC Curve Analysis of IAP as a Predictor of Mortality.

ROC analysis demonstrated that the predictive accuracy of IAP for mortality increased progressively over time, with the highest discriminative performance observed on day 7, followed by day 5 (Table 7, Figure 3).

### 3.10. Serial IAP Measurements

**Figure 4:**
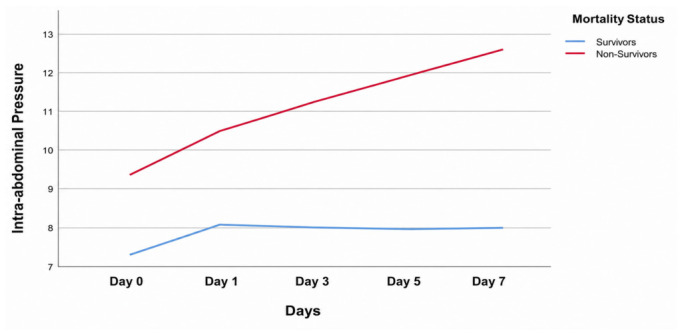
Daily Course of Intra-abdominal Pressure in Survivors and Non-Survivors.

Descriptive analysis of serial IAP measurements showed a progressive increase in IAP among non-survivors, whereas IAP values remained relatively stable in survivors after the initial ICU period. This divergence became more pronounced over time (Figure 4).

### 3.11. Association of Abdominal Perfusion Pressure with AKI and Mortality

Additional multivariable logistic regression analyses were performed to evaluate APP as an independent predictor of AKI and 90-day mortality. APP was entered as a continuous variable, with effect estimates expressed per 10-mmHg increase, and the models were adjusted for age, sex, Charlson Comorbidity Index, and APACHE II score. In the univariable analysis, higher APP was associated with lower odds of AKI (OR 0.69, 95% CI 0.51–0.93; *p* = 0.015). After multivariable adjustment, this association was attenuated and did not reach statistical significance (adjusted OR 0.70, 95% CI 0.48–1.01; *p* = 0.055). APP was not independently associated with 90-day mortality (adjusted OR 0.99, 95% CI 0.72–1.37; *p* = 0.956). The AKI and mortality models demonstrated acceptable discrimination, with AUC values of 0.837 and 0.776, respectively, and showed no evidence of poor calibration according to the Hosmer–Lemeshow test (Table 8).

## 4. Discussion

In this study, the impact of IAH on clinical outcomes in critically ill adult patients admitted to the ICU was comprehensively evaluated. Our findings demonstrate that IAH has a high prevalence and is strongly associated with organ dysfunction, positive fluid balance, and increased mortality. Furthermore, the higher AUC values observed for later IAP measurements suggest that serial IAP assessment may provide useful prognostic information.

In our cohort, the prevalence of IAH was 54%, which is higher than the 30–49% range reported in the literature [15,16,17]. This discrepancy may be attributed to the advanced age, high comorbidity burden, and severe clinical condition of our patient population. The observation that IAH can develop not only at ICU admission but also during follow-up further supports the need for serial monitoring rather than reliance on a single measurement.

When factors associated with the development of IAH were evaluated, advanced age, male sex, higher body mass index, increased comorbidity burden, presence of sepsis, positive fluid balance, and higher disease severity scores (SOFA and APACHE II) were identified as significant determinants. These findings are consistent with the literature and support the role of obesity and reduced abdominal compliance in the pathogenesis of IAH [18,19]. Similarly, aggressive fluid resuscitation in critically ill patients is known to contribute to increased IAP through intestinal edema and interstitial fluid accumulation [20].

Considering the multisystem effects of IAH, the renal system appears to be among the earliest affected organs [21]. In our study, the frequency of AKI occurrence was significantly higher in patients with IAH. Moreover, the positive correlation between IAP and serum urea and creatinine levels supports the detrimental effect of IAH on renal perfusion. This relationship can be explained by increased renal vascular resistance and decreased glomerular filtration rate. The higher requirement for renal replacement therapy in patients with IAH further underscores the clinical significance of this association. Our findings reinforce the importance of early recognition and prevention of renal injury, since deterioration in kidney function is associated not only with adverse short-term outcomes but also with long-term cardiovascular complications and increased mortality, highlighting the broad clinical consequences of renal dysfunction [22].

Although positive admission fluid balance was significantly associated with IAH in our cohort, only admission fluid balance was available for analysis. Therefore, we were unable to evaluate the impact of dynamic cumulative fluid balance throughout the ICU stay on the development of IAH, AKI, and mortality. Given that progressive fluid accumulation may contribute to intestinal edema, elevated intra-abdominal pressure, impaired abdominal organ perfusion, and subsequent organ dysfunction, serial cumulative fluid balance measurements would likely provide a more comprehensive understanding of these pathophysiological relationships. Future prospective studies incorporating longitudinal fluid balance assessments are warranted to further clarify these associations.

Our findings also indicate a strong association between IAH and mortality. Patients who developed IAH had significantly higher mortality rates, consistent with previous studies [23,24,25]. The observed association between IAH and mortality in multivariable analyses supports the potential prognostic relevance of IAH in critically ill patients. Additionally, the independent effect of the Charlson Comorbidity Index on mortality emphasizes the impact of overall disease burden. It should be emphasized that the optimal cut-off values identified by ROC analysis should not be interpreted as alternative diagnostic thresholds for intra-abdominal hypertension. The established diagnostic criterion for IAH remains an IAP ≥ 12 mmHg according to the WSACS definition. In contrast, the ROC-derived cut-off values simply represent the thresholds that provided the best discrimination for 90-day mortality in this specific cohort. These lower values likely reflect the fact that mortality risk increases progressively across the continuum of IAP rather than only after the diagnostic threshold for IAH is reached. To further evaluate the robustness of these findings, exploratory interaction analyses were performed according to sepsis, mechanical ventilation, age, and BMI. No significant effect modification was observed for the associations of IAH with either AKI or 90-day mortality (all *p* for interaction > 0.05; Supplementary Table S1). These findings suggest that the observed associations of IAH with adverse outcomes were generally consistent across these clinically relevant patient characteristics. However, because the study was not specifically powered for interaction analyses, these exploratory findings should be interpreted with caution.

When APP was evaluated, it was significantly lower in patients with IAH. In additional analyses, higher APP was associated with lower odds of AKI in the univariable model; however, this association was attenuated after adjustment for age, sex, comorbidity burden, and illness severity and narrowly failed to reach statistical significance. APP was also not independently associated with 90-day mortality. These findings suggest that reduced abdominal perfusion may reflect the combined effects of hemodynamic impairment, elevated IAP, comorbidity, and overall disease severity rather than acting as an independent prognostic determinant. Nevertheless, the direction and magnitude of the association with AKI warrant further evaluation in larger adequately powered cohorts.

Another notable finding of our study was the shorter ICU length of stay in patients with IAH. Although previous studies have generally reported prolonged ICU stays in such patients, this discrepancy may be explained by the higher mortality observed in our cohort. Consistently, survival analysis demonstrated significantly shorter survival in patients with IAH. Furthermore, the progressive increase in IAP observed in non-survivors highlights the prognostic importance of serial measurements in IAP [26,27,28].

Nevertheless, these findings should be interpreted in light of the potential survivor bias inherent in the study design. Because patients who died within the first seven days after ICU admission were excluded from the analytical cohort, analyses involving day 5 and day 7 IAP measurements were necessarily restricted to patients who survived long enough to undergo the scheduled serial assessments. Consequently, the higher AUC values observed at later time points may partly reflect the characteristics of this selected survivor population rather than indicating genuinely superior prognostic performance of later IAP measurements. Therefore, the apparent superiority of day 5 and day 7 IAP measurements should be interpreted cautiously and regarded as hypothesis-generating rather than definitive evidence of superior prognostic performance. Similarly, survival analyses may also have been influenced by the exclusion of patients who died during the first seven days and should therefore be interpreted in the context of this potential selection bias.

In conclusion, this study demonstrates that intra-abdominal hypertension is highly prevalent in critically ill patients, is significantly associated with impaired renal function and reduced survival, and is independently associated with mortality. These findings emphasize the importance of regular and serial monitoring of IAP in ICU patients for early identification of high-risk individuals and timely implementation of appropriate interventions.

Beyond the hemodynamic effects of intra-abdominal hypertension, emerging evidence suggests that dysregulation of mammalian target of rapamycin (mTOR) signaling may contribute to the link between elevated intra-abdominal pressure and acute kidney injury. Experimental studies have demonstrated that mTOR regulates autophagy, inflammation, oxidative stress, mitochondrial homeostasis, and tubular epithelial cell survival, all of which play central roles in the pathogenesis of AKI [29]. Although mTOR activity was not evaluated in the present study, the strong association between IAH, AKI, and mortality raises the possibility that mTOR-mediated pathways may contribute to these adverse outcomes, consistent with growing evidence linking elevated intra-abdominal pressure to renal injury [30]. Future translational studies integrating serial IAP measurements with molecular biomarkers of mTOR activation are warranted to further clarify these mechanisms [30].

The main limitations of this study include its single-center design, relatively small sample size, and the exclusion of certain high-risk patient populations. In addition, patients who died within the first 7 days of ICU admission were excluded because completion of the predefined serial IAP measurements (days 0, 1, 3, 5, and 7) was required for inclusion in the primary analyses. This exclusion may have introduced survivor bias, as patients who died early may have represented the most severely ill subgroup. Consequently, analyses involving day 5 and day 7 IAP measurements were restricted to patients who survived long enough to undergo these assessments, and the higher predictive performance observed at these later time points may have been influenced by this selection process rather than reflecting a true improvement in the prognostic performance of later IAP measurements. Accordingly, comparisons of prognostic performance across different time points should be interpreted cautiously. Although the initial full models had relatively low EPV ratios, the final parsimonious models achieved substantially higher EPV values and demonstrated minimal optimism after bootstrap internal validation. Nevertheless, residual confounding and some degree of model instability cannot be completely excluded. Therefore, the generalizability of the findings is limited, and larger multicenter studies with comprehensive longitudinal data collection are warranted to validate our findings.

## 5. Conclusions

This prospective cohort study demonstrates that IAH is common in critically ill patients and is significantly associated with increased mortality. Our findings suggest that IAH was independently associated with adverse outcomes and may contribute to multi-organ dysfunction.

Serial IAP measurements were associated with mortality in this cohort. However, the apparently greater prognostic performance of later measurements should be interpreted cautiously because of the potential survivor bias inherent in the study design.

However, given the single-center design and limited sample size, these findings should be interpreted as hypothesis-generating.

## Figures and Tables

**Table 1:** Demographic and Clinical Characteristics of Patients with Normal IAP and IAH.

Parameter	Normal IAP (*n* = 46)	IAH (*n* = 54)	*p*-Value
Age (years), mean ± SD	67.3 ± 16.8	74.6 ± 12.1	0.016 *
Sex, *n* (%)			
Male	19 (41.3)	37 (68.5)	0.006 **
Female	27 (58.7)	17 (31.5)	
Height (cm), mean ± SD	165.3 ± 8,7	165.6 ± 24,4	0.126 ***
Weight (kg), mean ± SD	75.0 ± 14.3	84.1 ± 15.6	0.007 ***
BMI (kg/m^2^), mean ± SD	27.5 ± 4.8	29.5 ± 5.0	0.042 ***
Comorbidity, *n* (%)			
Yes	37 (80.4)	53 (98.1)	0.005 **
No	9 (19.6)	1 (1.9)	
CCI, mean ± SD	4.6 ± 2.8	7.5 ± 2.9	<0.001 ***
Sepsis, *n* (%)			
Yes	15 (32.6)	38 (70.4)	<0.001 **
No	31 (67.4)	16 (29.6)	
Admission diagnosis, *n* (%)			
AKI	0 (0.0) ^a^	8 (14.8 ^b^)	0.002 **
Pneumonia	10 (21.7) ^a^	12 (22.2) ^a^	
Stroke	22 (47.8) ^a^	21 (38.9) ^a^	
Others	14 (30.4) ^a^	13 (24.1) ^b^	
ICU LOS (days), mean ± SD	23.1 ± 18.3	17.1 ± 18.0	0.008 ***
SBP (mmHg), mean ± SD	123.0 ± 20.3	123.3 ± 22.0	0.937 *
DBP (mmHg), mean ± SD	64.3 ± 13.9	62.2 ± 14.9	0.482 *
MAP (mmHg), mean ± SD	83.5 ± 12.1	82.0 ± 13.5	0.541 *
Volume status (Day 0), *n* (%)			
Euvolemic	35 (76.1) ^a^	28 (51.9) ^b^	0.041 **
Hypovolemic	5 (10.9) ^a^	10 (18.5) ^a^	
Hypervolemic	6 (13.0) ^a^	16 (29.6) ^b^	
SOFA score, mean ± SD	5.7 ± 2.7	8.7 ± 2.9	<0.001 ***
APACHE II score, mean ± SD	19.5 ± 7.1	28.2 ± 9.1	<0.001 *
Mechanical ventilation, *n* (%)			
Yes	33 (71.7)	47 (87.0)	0.057 **
No	13 (28.3)	7 (13.0)	
Inotropic support, *n* (%)			
Yes	25 (54.3)	44 (81.5)	0.003 **
No	21 (45.7)	10 (18.5)	
AKI occurrence, *n* (%)			
Yes	7 (15.2)	46 (85.2)	<0.001 **
No	39 (84.8)	8 (14.8)	
HD requirement, *n* (%)			
Yes	0 (0.0)	14 (25.9)	<0.001 **
No	46 (100.0)	40 (74.1)	
HD modality, *n* (%)			
CRRT	-	13 (92.9)	**-**
Conventional	-	1 (7.1)	**-**
Mortality, *n* (%)			
Yes	15 (32.6)	42 (77.8)	<0.001 **
No	31 (67.4)	12 (22.2)	
IAP Day 0 (mmHg), mean ± SD	5.4 ± 2.4	11.1 ± 3.9	<0.001 *
IAP Day 1 (mmHg), mean ± SD	6.2 ± 2.3	12.3 ± 3.5	<0.001 *
IAP Day 3 (mmHg), mean ± SD	6.6 ± 2.1	12.7 ± 3.5	<0.001 ***
IAP Day 5 (mmHg), mean ± SD	7.0 ± 2.3	13.0 ± 3.3	<0.001 ***
IAP Day 7 (mmHg), mean ± SD	7.3 ± 2.2	13.5 ± 3.6	<0.001 ***
ACS, *n* (%)			
Yes	0 (0.0)	4 (7.4)	0.122 **
No	46 (100.0)	50 (92.6)	
APP (mmHg), mean ± SD	78.7 ± 12.9	70.4 ± 14.5	<0.001 ***

BMI, body mass index; MAP, mean arterial pressure; SBP; Systolic blood pressure; DBP, Diastolic blood pressure; HD, hemodialysis; CRRT, continuous renal replacement therapy; SD, standard deviation; CCI, Charlson Comorbidity Index; LOS, Length of stay; Statistical tests: * Student’s *t*-test; ** chi-square test; *** Mann–Whitney U test. Note: Different superscript letters within a row indicate statistically significant differences between groups.

**Table 2:** Baseline Laboratory Parameters of Survivors and Non-Survivors.

Parameter	Survivors	Non-Survivors	*p*-Value
Urea (mg/dL)	63.4 ± 51.5	80.1 ± 60.9	0.020
Creatinine (mg/dL)	1.2 ± 0.8	1.6 ± 1.5	0.050
AST (U/L)	203.1 ± 573.7	152.9 ± 490.1	0.035
LDH (U/L)	405.4 ± 419.5	521.1 ± 776.8	0.030
Procalcitonin (ng/mL)	4.6 ± 16.0	7.1 ± 17.7	0.014
pH	7.4 ± 0.1	7.3 ± 0.1	0.031
Lactate (mmol/L)	2.4 ± 2.4	2.9 ± 2.2	0.042

**Table 3:** Parameters Significantly Correlated with IAP.

Parameter	Correlation Coefficient (r)	*p*-Value
BMI (kg/m^2^)	0.221	0.027
Charlson Comorbidity Index	0.332	0.001
APACHE II Score	0.397	<0.001
Urea (mg/dL)	0.508	<0.001
Creatinine (mg/dL)	0.672	<0.001
Albumin (g/L)	−0.296	0.003
AST (U/L)	0.244	0.015
ALT (U/L)	0.199	0.048
LDH (U/L)	0.199	0.048
Procalcitonin (ng/mL)	0.410	<0.001
pH	−0.299	0.003
H_2_CO_3_ (mmol/L)	−0.300	0.002
Lactate (mmol/L)	0.310	0.002

**Table 4:** Demographic and Clinical Characteristics of Patients with IAH at Baseline and During Follow-up.

Parameter	Normal IAP (*n* = 46)	IAH Present at Admission (*n* = 24)	IAH Developing During Follow-Up (*n* = 30)	*p*-Value
Age (years), mean ± SD	67.3 ± 16.8	75.0 ± 11.5	74.3 ± 12.7	0.048 *
Sex, *n* (%)				
Male	19 (41.3) ^a^	21 (87.5) ^b^	16 (53.3) ^a^	0.001 **
Female	27 (58.7) ^a^	3 (12.5) ^b^	14 (46.7) ^a^	
Height (cm), mean ± SD	165.3 ± 8.7	163.3 ± 35.8	167.4 ± 8.2	0.245 ***
Weight (kg), mean ± SD	75.0 ± 14.3 ^a^	86.4 ± 20.4 ^b^	82.3 ± 10.4 ^a,b^	0.008 *
BMI (kg/m^2^), mean ± SD	27.5 ± 4.8	29.4 ± 5.6	29.5 ± 4.6	0.135 *
Comorbidity, *n* (%)				
Yes	37 (80.4) ^a^	24 (100.0) ^a^	29 (96.7) ^a^	0.013 **
No	9 (19.6) ^a^	0 (0.0) ^a^	1 (3.3) ^a^	
CCI, mean ± SD	4.6 ± 2.8 ^a^	8.1 ± 2.5 ^b^	7.0 ± 3.1 ^b^	<0.001 ***
Sepsis, *n* (%)				
Yes	15 (32.6) ^a^	16 (66.7) ^b^	22 (73.3) ^b^	0.001 **
No	31 (67.4) ^a^	8 (33.3) ^b^	8 (26.7) ^b^	
Admission diagnosis, *n* (%)				
AKI	0 (0.0) ^a^	7 (29.2) ^b^	1 (3.3) ^a^	<0.001
Pneumonia	10 (21.7) ^a^	9 (37.5) ^a^	12 (40.0) ^a^	
Stroke	22 (47.8) ^a^	2 (8.3) ^b^	11 (36.7) ^a^	
Others	14 (30.4) ^a^	6 (25.0) ^a^	6 (20.0) ^a^	
ICU LOS (days), mean ± SD	23.1 ± 18.3 ^a^	20.8 ± 23.4 ^a,b^	14.1 ± 11.6 ^b^	0.019 ***
SBP (mmHg), mean ± SD	123.0 ± 20.3	124.4 ± 19.3	122.4 ± 24.2	0.941 *
DBP (mmHg), mean ± SD	64.3 ± 13.9	61.8 ± 15.2	62.6 ± 14.9	0.769 *
MAP (mmHg), mean ± SD	83.5 ± 12.1	81.5 ± 12.2	82.4 ± 14.6	0.804 *
Volume status (Day 0), *n* (%)				
Euvolemic	35 (76.1) ^a^	7 (29.2) ^b^	21 (70.0) ^a^	<0.001 **
Hypovolemic	5 (10.9) ^a^	4 (16.7) ^a^	6 (20.0) ^a^	
Hypervolemic	6 (13.0) ^a^	13 (54.2) ^b^	3 (10.0) ^a^	
SOFA score, mean ± SD	5.7 ± 2.7 ^a^	8.7 ± 3.3 ^b^	8.8 ± 2.6 ^b^	<0.001 ***
APACHE II score, mean ± SD	19.5 ± 7.1 ^a^	27.9 ± 7.2 ^b^	28.3 ± 10.4 ^b^	<0.001 *
Mechanical ventilation, *n* (%)				
Yes	33 (71.7)	20 (83.3)	27 (90.0)	0.140 **
No	13 (28.3)	4 (16.7)	3 (10.0)	
Inotropic support, *n* (%)				
Yes	25 (54.3) ^a^	19 (79.2) ^a,b^	25 (83.3) ^b^	0.013 **
No	21 (45.7) ^a^	5 (20.8) ^a,b^	5 (16.7) ^b^	
AKI occurrence, n (%)				
Yes	7 (15.2) ^a^	20 (83.3) ^b^	26 (86.7) ^b^	<0.001 **
No	39 (84.8) ^a^	4 (16.7) ^b^	4 (13.3) ^b^	
HD requirement, *n* (%)				
Yes	0 (0.0) ^a^	8 (33.3) ^b^	6 (20.0) ^b^	<0.001 **
No	46 (100.0) ^a^	16 (66.7) ^b^	24 (80.0) ^b^	
HD modality, *n* (%)				
CRRT	-	7 (87.5)	6 (100.0)	1.000 *
Conventional	-	1 (12.5)	0 (0.0)	
Mortality, *n* (%)				
Yes	15 (32.6) ^a^	17 (70.8) ^b^	25 (83.3) ^b^	<0.001 **
No	31 (67.4) ^a^	7 (29.2) ^b^	5 (16.7) ^b^	
IAP Day 0 (mmHg), mean ± SD	5.4 ± 2.4 ^a^	14.6 ± 2.8 ^b^	8.3 ± 2.0 ^c^	<0.001 ***
IAP Day 1 (mmHg), mean ± SD	6.2 ± 2.3 ^a^	15.1 ± 2.6 ^b^	10.0 ± 2.2 ^c^	<0.001 ***
IAP Day 3 (mmHg), mean ± SD	6.6 ± 2.1 ^a^	14.3 ± 3.9 ^b^	11.3 ± 2.5 ^b^	<0.001 ***
IAP Day 5 (mmHg), mean ± SD	7.0 ± 2.3 ^a^	14.0 ± 4.0 ^b^	12.2 ± 2.3 ^b^	<0.001 ***
IAP Day 7 (mmHg), mean ± SD	7.3 ± 2.2 ^a^	13.5 ± 4.7 ^b^	13.5 ± 2.5 ^b^	<0.001 ***
ACS, *n* (%)				
Yes	0 (0.0) ^a^	4 (16.7) ^b^	0 (0.0) ^a,b^	0.003 **
No	46 (100.0) ^a^	20 (83.3) ^b^	30 (100.0) ^a,b^	
APP (mmHg), mean ± SD	78.7 ± 12.9 ^a^	65.8 ± 12.7 ^b^	74.0 ± 15.1 ^a,b^	<0.001 ***

BMI, body mass index; MAP, mean arterial pressure; SBP; Systolic blood pressure; DBP, Diastolic blood pressure; HD, hemodialysis; CRRT, continuous renal replacement therapy; SD, standard deviation; CCI, Charlson Comorbidity Index; LOS, Length of stay; Statistical tests: * Student’s *t*-test; ** chi-square test; *** Mann–Whitney U test. Note: Different superscript letters within a row indicate statistically significant differences between groups.

**Table 5:** Parsimonious Multivariable Logistic Regression Model for Factors Associated with IAH.

Parameter	β	OR	95% CI	*p*-Value
SOFA score	0.429	1.536	1.174–2.009	0.002
AKI	3.382	29.441	8.497–102.003	<0.001

OR, odds ratio; CI, confidence interval; AKI, acute kidney injury; SOFA, Sequential Organ Failure Assessment. Model performance: 54 events, 2 predictors, EPV = 27.0; AUC = 0.922; optimism-corrected AUC = 0.920; Hosmer–Lemeshow *p* = 0.296; bootstrap calibration slope = 0.938 (1000 resamples).

**Table 6:** Parsimonious Multivariable Cox Regression Model for Factors Associated with Mortality.

Parameter	B	SE	Wald	*p* Value	HR (95% CI)
Development of IAH	0.910	0.323	7.94	0.005	2.48 (1.32–4.68)
Charlson Comorbidity Index	0.139	0.046	8.92	0.003	1.15 (1.05–1.26)

HR, hazard ratio; CI, confidence interval; IAH, intra-abdominal hypertension. Model performance: 57 deaths, 2 predictors, EPV = 28.5; Harrell C-index = 0.736; optimism-corrected C-index = 0.733; bootstrap calibration slope = 0.973 (1000 resamples).

**Table 7:** ROC Analysis of Serial Intra-Abdominal Pressure Measurements for Prediction of Mortality.

IAP	AUC	*p*-Value	95% CI	Cut-Off Value (mmHg)	Sensitivity (%)	Specificity (%)
Day 0	0.626	0.031	0.517–0.736	7.5	61.4	58.1
Day 1	0.661	0.006	0.554–0.768	8.5	61.4	58.1
Day 3	0.713	<0.001	0.613–0.813	8.5	70.2	60.5
Day 5	0.789	<0.001	0.698–0.880	9.5	75.4	76.7
Day 7	0.813	<0.001	0.727–0.900	9.5	80.7	76.7

**Table 8:** Multivariable Logistic Regression Analyses Evaluating APP as a Predictor of AKI and 90-Day Mortality.

Outcome	APP Effect	Adjusted OR	95% CI	*p*-Value
AKI	Per 10-mmHg increase	0.70	0.480–1.01	0.055
90-day mortality	Per 10-mmHg increase	0.99	0.720–1.370	0.956

Models were adjusted for age, sex, Charlson Comorbidity Index, and APACHE II score. APP, abdominal perfusion pressure; AKI, acute kidney injury; OR, odds ratio; CI, confidence interval.

## Data Availability

The data presented in this study are available on reasonable request from the corresponding author. The data are not publicly available due to privacy and ethical restrictions related to patient confidentiality.

## Supplementary Materials

The following supporting information can be downloaded at: https://www.mdpi.com/article/10.3390/jcm15145742/s1, Table S1: Exploratory Interaction Analyses Evaluating Effect Modification of the Associations of IAH with AKI and 90-Day Mortality.

## Abbreviations

The following abbreviations are used in this manuscript:

IAH	Intra-abdominal hypertension
IAP	Intra-abdominal pressure
APP	Abdominal perfusion pressure
ACS	Abdominal compartment syndrome
AKI	Acute kidney injury
ICU	Intensive care unit
SOFA	Sequential Organ Failure Assessment
APACHE	Acute Physiology and Chronic Health Evaluation
ROC	Receiver operating characteristic
