# Supplementary material for: Association of Serial Intra-Abdominal Pressure Measurements with Renal Outcomes and Mortality in Critically Ill Adults
Source: J Clin Med. 2026 Jul 22;15(14):5742. doi: 10.3390/jcm15145742 (PMC13412193; doi:10.3390/jcm15145742)
Supplement: Supplementary file 1 [file jcm-15-05742-s001.zip › jcm-4423322-supplementary.pdf]

Supplementary Table S1. Exploratory Interaction Analyses Evaluating Effect Modification of the Associations of IAH with AKI and 90-Day Mortality

**Outcome**	**Effect modifier**	**P for interaction**
AKI	Sepsis	0.435
90-day mortality	Sepsis	0.300
AKI	Mechanical ventilation	0.562
90-day mortality	Mechanical ventilation	0.811
AKI	Age	0.698
90-day mortality	Age	0.266
AKI	BMI	0.121
90-day mortality	BMI	0.149
